# Ca^2+^ signaling in plant manganese uptake: CPK21/23 kinases phosphorylate and activate manganese transporter NRAMP1

**DOI:** 10.1007/s44154-022-00067-w

**Published:** 2022-10-20

**Authors:** Chao-Feng Huang

**Affiliations:** National Key Laboratory of Plant Molecular Genetics, Shanghai Center for Plant Stress Biology, CAS Center for Excellence in Molecular Plant Sciences, Shanghai Institute of Plant Physiology and Ecology, Chinese Academy of Sciences, Shanghai, 200032 China

**Keywords:** Manganese deficiency, Ca^2+^ signaling, Ca^2+^-dependent protein kinase (CPK), NRAMP1 phosphorylation

## Abstract

This brief article highlights the results of Fu et al. (Proc Natl Acad Sci USA 119:e2204574119, 2022), who recently found that manganese (Mn) deficiency triggers long-lasting multicellular Ca^2+^ oscillations in the elongation zone (EZ) of Arabidopsis roots and revealed a Ca^2+^-CPK21/23-NRAMP1 axis as an important mechanism for plant tolerance and adaptation to low Mn.

The micronutrient manganese (Mn) plays essential roles in diverse biological processes, including photosynthesis, antioxidant defense and cell wall biosynthesis (Marschner 2012; Yang et al. 2021). Mn bioavailability in soils is mainly determined by soil pH and water content. Mn deficiency usually occurs in dry and alkaline soils, whereas Mn toxicity happens in well-watered and acidic soils (Schmidt et al. 2016). Mn transporters are crucial for plants to adapt to the large fluctuations of Mn concentration in the soils.

Calcium ion (Ca^2+^) is a critical signaling transducer in all eukaryotic organisms. In plants, Ca^2+^ signals are decoded by two signaling networks, Ca^2+^-dependent protein kinases (CPKs) and the CBL-CIPK network composed of calcineurin-B-like Ca^2+^ sensor proteins (CBLs) and CBL-interacting protein kinases (CIPKs) (Asano et al. 2012; Kudla et al. 2018; Dong et al. 2021). Zhang et al. (2021) recently reported that excess Mn is able to trigger rapid and transient Ca^2+^ signals in Arabidopsis roots. These excess Mn-induced Ca^2+^ signals can be decoded by four Ca^2+^-dependent protein kinases, CPK4/5/6/11, which phosphorylate and activate the tonoplast-localized Mn transporter MTP8 that is critically required for Mn detoxification (Zhang et al. 2021). More recently, the same research group showed that CBL2/3-CIPK3/9/26 signaling network can also phosphorylate MTP8 but negatively regulate its activity (Ju et al. 2022). However, whether Ca^2+^ signaling plays an important role in plant response and tolerance to Mn deficiency remains unclear. This brief article highlights the recent results of Fu et al. (2022), who revealed a Ca^2+^-CPK21/23-NRAMP1 axis as an important mechanism for plant tolerance and adaptation to low Mn.

Previously, a group of researchers from Northwest A&F University (Yangling, Shaanxi, China) have used aequorin reporter to detect Ca^2+^ signals in response to excess Mn (Zhang et al. 2021). Nevertheless, this aequorin-based investigation provided limited spatiotemporal resolution of the Ca^2+^ signals. In the study of the same research group (Fu et al. 2022), they employed an ultrasensitive ratiometric Ca^2+^ reporter protein GCaMP6f-mCherry that combines the superior dynamic range and temporal accuracy of GCaMP6f with ratiometric data obtained by normalization through mCherry emission monitoring to detect spatiotemporal dynamic Ca^2+^ signals in response to low Mn stress, and discovered that elongation zone (EZ) of Arabidopsis roots exhibited oscillatory Ca^2+^ signals after exposure to low Mn. These Ca^2+^ oscillations initiated 126 ± 4 min after exposure to low Mn and displayed a frequency of 31 ± 6 min and amplitude of 0.14 ± 0.04 Δ*R*/*R*_0_ on average. They also observed a remarkable differentiation of Ca^2+^ oscillation formation in regard to its longitudinal and transversal dimensions. Together, their results revealed an interesting finding that Mn deficiency induces long-lasting multicellular Ca^2+^ oscillations in the EZ of Arabidopsis roots, although whether these Ca^2+^ oscillations are triggered directly by Mn deficiency or indirectly by Mn deficiency-induced other changes such as growth inhibition remains further clarification.

To identify components potentially decoding the oscillating Ca^2+^ signals in response to Mn deficiency, Fu et al. (2022) performed bimolecular fluorescence complementation (BiFC) assays to search CPKs that can interact with the high-affinity Mn uptake transporter NRAMP1. As a result, they identified CPK21 and CPK23, which strongly interact with NRAMP1, as well as four other NRAMP1-interacting CPKs, CPK4 to CPK6 and CPK11. To ascertain which CPKs are important for regulating NRAMP1 function in vivo, they were committed to generate a suite of *cpk* mutants and mutant combinations and then examined which mutants showed impaired plant growth under Mn deficiency. Based on this phenotypic analysis, they revealed that CPK21/23 but not CPK4/5/6/11 are redundantly involved in the regulation of low Mn tolerance. They therefore further carried out protein interaction assays to confirm that CPK21 and CPK23 interact directly with NRAMP1 both in vitro and in vivo.

Next, by in vitro kinase assays, Fu et al. (2022) identified S20 and T498 of NRAMP1 as primary target sites of CPK21/23 phosphorylation, although S22 may also contribute to CPK21/23-mediated NRAMP1 phosphorylation. They then employed heterologous yeast complementation assays to determine the effect of S20/22 or T498 phosphorylation on NRAMP1 function and the results revealed that phosphorylation of T498 instead of S20/22 is critical for the Mn transport function of NRAMP1. Since a previous report showed that the phosphorylation status of S20/22/24 in NRAMP1 influences its plasma membrane retention under excess Mn conditions (Castaings et al. 2021), Fu et al. (2022) also examined the effect of T498 phosphorylation status on NRAMP1 subcellular localization and protein accumulation and their results revealed that T498 phosphorylation does not influence NRAMP1 localization and abundance. To convince the importance of T498 phosphorylation for NRAMP1 function in vivo, they conducted *nramp1* complementation assays and persuasively demonstrated that NRAMP1^T498^ phosphorylation is required for plant high-affinity Mn uptake and low Mn tolerance.

Finally, Fu et al. (2022) demonstrated that Mn deficiency is able to trigger NRAMP1^T498^ phosphorylation in plants, which relies on the presence of CPK21/23, and overexpression of *CPK23* increases plant tolerance to low Mn via the regulation of NRAMP1. Overall, their results revealed a key Ca^2+^ signaling mechanism for plant tolerance to Mn deficiency. In this mechanism, Mn deficiency triggers Ca^2+^ oscillations in the root EZ, which activate CPK21/23 kinases that promote NRAMP1 phosphorylation at Thr498 site, and the NRAMP1^T498^ phosphorylation increases its transport activity and consequently enhances plant Mn uptake and tolerance to Mn deficiency. Thus, the study by Fu et al. (2022) not only provides new insights into how Ca^2+^ signaling modulates plant Mn uptake but also provides a new tool to enhance plant tolerance to Mn deficiency by engineering NRAMP1 phosphorylation.

## Data Availability

Not applicable.

## Abbreviations

Mn: Manganese
CPK: Ca^2+^-dependent protein kinase
CBL: Calcineurin-B-like Ca^2+^ sensor protein
CIPK: CBL-interacting protein kinase
BiFC: Bimolecular fluorescence complementation
EZ: Elongation zone
NRAMP1: Natural Resistance Associated Macrophage Protein 1
